# A six-metabolite signature characterizes metabolically unhealthy obesity and reveals hidden metabolic risk within metabolically healthy obesity

**DOI:** 10.3389/fendo.2026.1910610

**Published:** 2026-09-11

**Authors:** Pan Gao, Meifang Liang, Binfeng Tang, Jingyi Li, Rui Mao, Zhonghui Feng, Tongtong Zhang, Yanjun Liu

**Affiliations:** 1 Obesity and Metabolism Medicine-Engineering Integration Laboratory, Department of General Surgery, The Third People’s Hospital of Chengdu, Chengdu, Sichuan, China; 2 The Center of Obesity and Metabolic Diseases, Department of General Surgery, The Third People’s Hospital of Chengdu, Chengdu, Sichuan, China; 3 College of Life Science and Engineering, Southwest Jiaotong University, Chengdu, Sichuan, China; 4 College of Medicine, Southwest Jiaotong University, Chengdu, Sichuan, China; 5 Department of Dermatology, Xiangya Hospital, Central South University, Changsha, China

**Keywords:** metabolic risk, metabolically healthy obesity, metabolically unhealthy obesity, metabolomics, proteomics

## Abstract

**Background/objectives:**

Metabolically healthy obesity (MHO) is commonly defined by the absence of metabolic syndrome-related abnormalities despite obesity. However, conventional clinical definitions may overlook substantial metabolic heterogeneity and hidden cardiometabolic risk. We aimed to identify metabolomic signatures distinguishing MHO from metabolically unhealthy obesity (MUHO), evaluate their discriminatory performance, and determine whether metabolomic profiling could further characterize heterogeneity within conventionally defined MHO.

**Methods:**

We analyzed 13215 UK Biobank adults with obesity and available clinical biomarker and NMR-based metabolomic data. Metabolic health was defined using triglycerides, HDL cholesterol, hypertension, fasting glucose, type 2 diabetes, and lipid-lowering medication use. Univariable logistic regression and LASSO regression were used for metabolite selection. Logistic regression and XGBoost models were developed using clinical variables, metabolomic markers, and their combination. A weighted metabolic signature score was applied within the MHO group to characterize cross-sectional metabolic and clinical heterogeneity, and proteomic analyses were performed in approximately 1408 participants.

**Results:**

A six-metabolite signature comprising HDL_size, S_HDL_CE, XL_HDL_TG, GlycA, M_VLDL_C, and Omega_3 was selected. The combined clinical-metabolomic model showed better discrimination than clinical variables alone in the test set, with AUCs of 0.78 and 0.69, respectively. Within MHO, higher metabolomic score was associated with higher triglycerides, HbA1c, waist-to-hip ratio, lower HDL cholesterol, and greater metabolic and cardiovascular comorbidity burden. Proteomic analyses identified 10 metabolite-associated core proteins implicating lipoprotein remodeling, adipokine signaling, inflammation, and vascular-related pathways.

**Conclusions:**

A six-metabolite signature distinguished MHO from MUHO and revealed hidden metabolic risk within conventionally defined MHO, provides a metabolomic framework for refining obesity phenotyping and warrants further validation before clinical translation.

## 1 Introduction

Obesity is a major global health challenge and a key driver of type 2 diabetes (T2D), hypertension, non-alcoholic fatty liver disease (NAFLD), and cardiovascular disease (1, 2). However, obesity is biologically heterogeneous, and individuals with similar body mass index (BMI) may differ substantially in insulin sensitivity, lipid metabolism, inflammatory status, and long-term clinical outcomes (3, 4). This heterogeneity has led to the distinction between metabolically healthy obesity (MHO), commonly defined as obesity with few or no major metabolic syndrome-related abnormalities, and metabolically unhealthy obesity (MUHO), characterized by overt metabolic dysfunction (5). Although MHO is generally associated with lower risk than MUHO, its clinical significance remains debated. Evidence from prospective cohorts demonstrated that MHO is associated with elevated risks of T2D, cardiovascular disease, heart failure, and mortality compared with metabolically healthy non-obesity (6, 7). Thus, MHO may represent a heterogeneous and dynamic phenotype rather than a stable low-risk state.

Current definitions of MHO and MUHO are mainly based on metabolic syndrome-related clinical indicators, including triglycerides, HDL cholesterol, blood pressure, fasting glucose, and related medication use (8). These criteria are clinically practical and standardized, but they may not fully capture the molecular heterogeneity underlying metabolic health, particularly among individuals classified as MHO. Previous metabolomic studies have reported differences between metabolically healthy and unhealthy obesity in lipoprotein subclasses, amino acids, fatty acids, and lipids supporting the value of metabolomics for characterizing obesity-related metabolic heterogeneity (9, 10). However, many prior studies were limited by modest sample size, restricted metabolite coverage, or insufficient integration with predictive modeling and biological interpretation. At present, it remains unclear whether metabolomic signatures distinguishing MHO from MUHO can identify hidden metabolic risk and uncover clinically relevant heterogeneity within individuals conventionally classified as MHO.

Large-scale multi-omics resources now provide an opportunity to characterize obesity-related metabolic heterogeneity with greater biological resolution. In the UK Biobank, nuclear magnetic resonance (NMR) metabolomics captures diverse circulating biomarkers, including lipoprotein subclasses, lipid composition, fatty acids, amino acids, and inflammatory markers (11, 12). These data enable the development of concise and interpretable metabolomic signatures for distinguishing MHO from MUHO. In addition, proteomics can provide biological context for metabolite-based signatures by linking metabolic profiles to pathways involving lipoprotein transport, adipokine signaling, inflammation and vascular regulation (13). Integrating metabolomics and proteomics may therefore help distinguish statistical prediction markers from biologically coherent molecular networks.

In this study, we analyzed UK Biobank adults with obesity and available clinical biomarker and metabolomics data. We identified metabolomic markers associated with MHO and MUHO, constructed a six-metabolite signature to distinguish these phenotypes, and evaluated its predictive performance alone and with clinical variables using logistic regression and XGBoost. As an exploratory analysis, we applied the signature-derived risk score within the MHO group to assess hidden metabolic risk gradients. Proteomic data from a subset of participants were further used to explore protein-level correlates and biological pathways linked to the signature. We hypothesized that a concise metabolomic signature could distinguish MHO from MUHO and further reveal metabolic heterogeneity within conventionally defined MHO.

## 2 Materials and methods

### 2.1 Study population

Participants were selected from the UK Biobank. A total of 61730 individuals had available clinical biomarker and NMR-based metabolomics data (14). Obesity was defined as a BMI ≥30 kg/m². Of these participants, 47,153 with a BMI<30 kg/m² were excluded. Among the remaining 14,577 participants with obesity, 1,362 were excluded because the available clinical information was insufficient to determine MHO or MUHO status. Missingness reflected the availability of measurements in the UK Biobank rather than investigator-controlled selection. Complete-case analysis was performed, and no multiple imputation was used. The final analytic cohort included 13,215 participants. Metabolic health status was classified using metabolic syndrome-related indicators, including triglycerides, high-density lipoprotein cholesterol (HDL-C), blood pressure, and fasting glucose (15, 16). Elevated triglycerides were defined as triglycerides≥1.7 mmol/L. Reduced HDL-C was defined as HDL-C< 1.03 mmol/L in men or<1.29 mmol/L in women. Elevated fasting glucose was defined as fasting glucose ≥ 5.6 mmol/L. Hypertension was defined according to blood pressure measurements, clinical diagnosis, or medication-related information. MHO was defined as obesity with no more than one metabolic abnormality and without T2D or lipid-lowering medication use. MUHO was defined as obesity with two or more metabolic abnormalities, or the presence of T2D or lipid-lowering medication use regardless of the number of metabolic abnormalities.

### 2.2 Clinical variables

Baseline variables included demographic characteristics, anthropometric indices, metabolic biomarkers, lifestyle factors, comorbidities, and medication use. Demographic and anthropometric variables included age, sex, BMI, waist circumference, and waist-to-hip ratio (WHR). Metabolic biomarkers included triglycerides, HDL cholesterol, fasting glucose, and glycated hemoglobin (HbA1c). Lifestyle factors included smoking status and alcohol consumption. Clinical comorbidities included T2D, hypertension, hyperlipidemia, NAFLD, cardiovascular disease (CVD), coronary heart disease (CHD), heart failure, sleep apnea, hyperuricemia, and myocardial infarction. Lipid-lowering medication use was also included. NAFLD was ascertained by combining linked hospital diagnostic records and self-reported medical history in the UK Biobank.

### 2.3 NMR-based metabolomic analysis

NMR-based metabolomics data were obtained from the UK Biobank. After quality control and harmonization, 154 circulating metabolites were included. These metabolites covered multiple metabolic domains, including lipoprotein subclasses, lipoprotein lipid composition, fatty acids, and other circulating metabolic biomarkers (17). Metabolite values were standardized before regression modeling. Univariable logistic regression was performed for each metabolite using MUHO status as the outcome. Odds ratios (OR), 95% confidence intervals (CI), P values, and false discovery rate-adjusted P values were calculated. Metabolites were classified as positively or negatively associated with MUHO according to the direction of regression coefficients. Least absolute shrinkage and selection operator (LASSO) regression with cross-validation was then used to reduce redundancy and identify candidate metabolomic markers. The potential metabolites were evaluated using a multidimensional selection strategy that considered (1): the magnitude of their LASSO coefficients and their individual statistical associations with metabolic health status (remain top 50%); (2) redundancy among metabolites, assessed using Pearson correlation analysis (remove |r|>0.6); (3) representation of distinct metabolic domains rather than inclusion of multiple highly correlated measurements reflecting the same biological process; and (4) biological relevance to lipid metabolism, inflammation, and obesity-related metabolic dysfunction.

### 2.4 Model construction

Three classification models were developed to distinguish MHO from MUHO, including clinical model, metabolomic model, and a combined clinical-metabolomic model. The primary clinical model included age, sex, and waist-to-hip ratio. To provide a stronger clinical comparator, we additionally constructed an expanded clinical model including age, sex, waist-to-hip ratio, BMI, smoking status, and alcohol consumption. Blood pressure, triglycerides, HDL-C, glucose, type 2 diabetes, and lipid-lowering medication use were not included because they contributed directly to the definition of MHO and MUHO, and their inclusion would introduce outcome–predictor circularity. The metabolomic model included the metabolites signature. The combined model included age, sex, WHR, and the selected metabolites. Participants were randomly divided into a training set containing 70% of the cohort and a test set containing the remaining 30%. Logistic regression models were fitted in the training set and evaluated in both training and test sets. Model discrimination was assessed using receiver operating characteristic curves. Differences in area under the curve (AUC) were evaluated using DeLong tests where appropriate (18, 19). Incremental predictive performance was assessed using net reclassification improvement and integrated discrimination improvement. Decision curve analysis was performed to evaluate clinical net benefit across threshold probabilities. To assess robustness across modeling strategies, XGBoost models were also developed using the same feature sets and evaluated in the training and test sets.

### 2.5 Metabolomic risk score

To explore heterogeneity within conventionally defined MHO, a weighted metabolomic risk score was calculated among MHO participants using the selected metabolite signature:


$$\text{Metabolomic}\;\text{risk}\;\text{score}\;=\;{\text{β}}_{0}+\;{\text{β}}_{1}*{\text{x}}_{1}+\;{\text{β}}_{2}*{\text{X}}_{2}+\ldots \;+\;{\text{β}}_{\text{i}}*{\text{X}}_{\text{i}}$$

where X_i_ represents the standardized value of each metabolite, β_0_ represents the intercept and β_i_ represents the corresponding regression coefficient from the prediction model. MHO participants were stratified into low-risk, intermediate-risk, and high-risk groups according to the distribution of the metabolomic risk score. Participants in the first quartile (Q1) were classified as the low-risk group, those in the second and third quartiles (Q2–Q3) as the intermediate-risk group, and those in the fourth quartile (Q4) as the high-risk group. Clinical phenotype, including triglycerides, HbA1c, HDL, WHR, hypertension, hyperlipidemia, NAFLD, sleep apnea, CVD, heart failure, and CHD were compared across score groups.

### 2.6 Proteomic analysis

Proteomic data were obtained from the UK Biobank Olink platform and were available for 1,408 participants in the analytic cohort. Missing protein measurements were imputed using the k-nearest neighbors algorithm. Protein abundance values were then standardized by z-score transformation before statistical analysis. Differential protein analysis was performed to identify proteins with altered abundance between MHO and MUHO. Differential proteins were defined using an adjusted P<0.05 and an absolute log2 fold change ≥ 0.5.

Pearson correlation analysis was performed between the six selected metabolites and the differential proteins. Multiple testing was controlled using the FDR method. Proteins with an absolute correlation coefficient >0.30 with at least one metabolite and an adjusted P<0.01 were defined as metabolite-associated core proteins. The |r| > 0.30 threshold was chosen to retain correlations of at least moderate magnitude while excluding weak associations. Because this analysis was exploratory, the primary protein–metabolite correlation analysis was not adjusted for clinical covariates. The resulting metabolite–protein network was visualized using Cytoscape v3.10.0 (20).

### 2.7 Sensitivity and subgroup analyses

Several sensitivity and subgroup analyses were conducted to assess the robustness and consistency of the main findings. First, because no universally accepted definition of MHO exists, we repeated the analyses using a stricter definition. Strict MHO was defined as obesity with no metabolic abnormalities, no type 2 diabetes, and no lipid-lowering medication use, whereas strict MUHO was defined as obesity with at least one metabolic abnormality, type 2 diabetes, or lipid-lowering medication use.

To examine whether phenotype discrimination was driven primarily by biological overlap between lipid-related metabolites and the lipid criteria used to define MUHO, we repeated the analysis after excluding HDL_size, S_HDL_CE, XL_HDL_TG, and M_VLDL_C, retaining GlycA and Omega_3 as non-lipid markers. In addition, an expanded clinical model including age, sex, waist-to-hip ratio, BMI, smoking status, and alcohol consumption was constructed and compared with the primary clinical model. The corresponding expanded clinical–metabolomic model was also evaluated.

To assess the potential influence of lipid-lowering therapy, we additionally excluded all participants reporting lipid-lowering medication use. The same prespecified six-metabolite panel and predictor sets were retained, and the clinical, metabolomic, and combined models were refitted using the same procedures as in the primary analysis. Internal validation and optimism correction were performed using bootstrap resamples. Model discrimination and calibration were evaluated using the optimism-corrected AUC, calibration intercept, calibration slope, and Brier score.

Finally, sex-stratified analyses were performed because of the marked difference in sex distribution between the MHO and MUHO groups and the known sex-related heterogeneity in obesity-associated metabolism. The combined model was evaluated separately in men and women in the training and test sets. Discrimination was assessed using the AUC with 95% confidence intervals, and calibration was assessed using the calibration intercept, calibration slope, and Brier score. Effect modification by sex was formally tested by including an interaction term between sex and the combined-model linear predictor in a logistic regression model.

### 2.8 Statistical analysis

Continuous variables are presented as mean ± standard deviation, and categorical variables as counts or percentages. Group comparisons were performed using Student’s t-test or nonparametric tests for continuous variables and chi-square tests for categorical variables, as appropriate. Logistic regression results are reported as OR with 95% CI. Multiple testing was controlled using the false discovery rate (FDR) method. All statistical tests were two-sided, and P< 0.05 were considered statistically significant. All statistical analyses were performed using R v4.2.0.

## 3 Results

### 3.1 Study population and baseline characteristics

**Figure 1:**
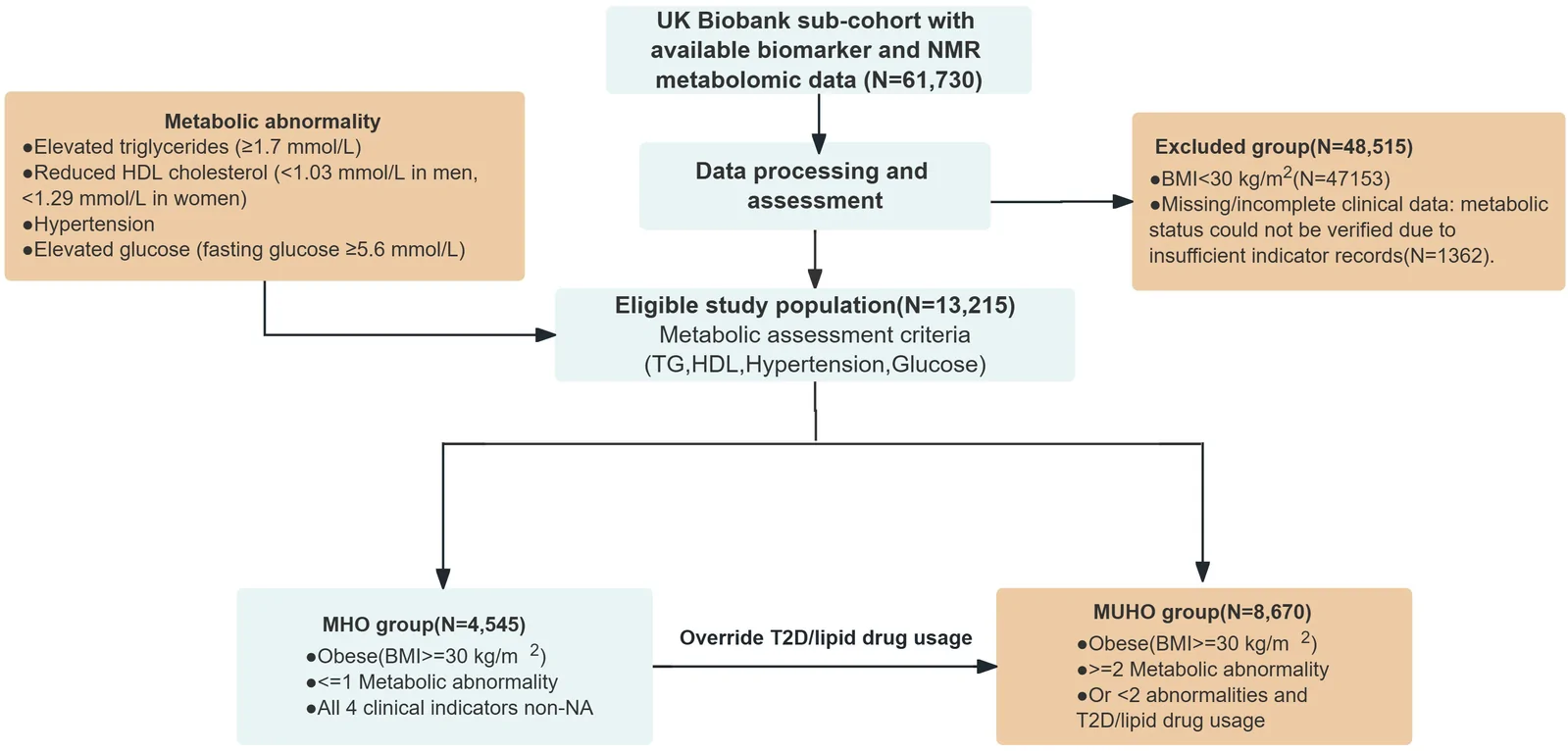
Study design and flow diagram of participant inclusion and exclusion.

Among UK Biobank participants with available clinical biomarker and NMR-based metabolomics data, individuals with BMI< 30 kg/m² or insufficient clinical information for metabolic health classification were excluded. The final analytic cohort comprised 13215 adults with obesity, including 4545 participants classified as MHO and 8670 classified as MUHO according to triglycerides, HDL cholesterol, hypertension status, and glucose-related criteria, with additional consideration of type 2 diabetes and lipid-lowering medication use (**Figure 1**).

Baseline characteristics differed markedly between the MHO and MUHO groups (**Table 1**). Participants with MUHO were older than those with MHO and had slightly higher BMI. However, the absolute BMI difference between groups was modest, whereas the difference in waist circumference was more pronounced, suggesting that metabolically unhealthy obesity was associated not only with greater overall adiposity but also with more adverse body fat distribution. Besides, participants with MUHO showed a substantially more unfavorable metabolic profile, including higher triglycerides, fasting glucose, and HbA1c levels, together with lower HDL. MUHO participants also had a higher burden of metabolic comorbidities, including type 2 diabetes, hypertension, hyperlipidemia, NAFLD, coronary heart disease, heart failure, and sleep apnea. Differences in sex distribution, smoking status, and alcohol consumption were also observed between groups. Overall, these findings indicate that MHO and MUHO represent clinically distinct obesity phenotypes despite broadly comparable BMI levels.

**Table 1:** Baseline characteristics of the study participants.

Characteristic	MHO	MUHO	*P* value
Participants, n	4545	8670	
Age (years), mean (s.d.)	54.77 (8.01)	58.01 (7.56)	<0.001
BMI (kg/m^2^)	33.34 (3.34)	34.32 (4.15)	<0.001
Waist circumference (cm)	101.40 (10.30)	107.32 (11.11)	<0.001
Triglycerides (mmol/L)	1.56 (0.78)	2.37 (1.17)	<0.001
HDL (mmol/L)	1.46 (0.30)	1.20 (0.28)	<0.001
Glucose (mmol/L)	4.98 (0.53)	5.67 (1.77)	<0.001
HbA1c (mmol/L)	34.65 (3.61)	40.52 (10.05)	<0.001
Sex, n (%)			<0.001
Male	2905 (63.9%)	4087 (47.1%)	
Female	1640 (36.1%)	4583 (52.9%)	
Smoking status, n (%)			<0.001
Never	2572 (56.8%)	4160 (48.2%)	
Ever	1960 (43.2%)	4475 (51.8%)	
Alcohol consumption, n (%)			<0.001
Never	1613 (35.5%)	3573 (41.2%)	
Regular	2931 (64.5%)	5092 (58.8%)	
T2D, n (%)			<0.001
Yes	0 (0.0%)	2738 (31.6%)	
No	4545 (100.0%)	5932 (68.4%)	
Hypertension, n (%)			<0.001
Yes	930 (20.5%)	6152 (71.0%)	
No	3615 (79.5%)	2518 (29.0%)	
Hyperlipidemia, n (%)			<0.001
Yes	476 (10.5%)	3057 (35.3%)	
No	4069 (89.5%)	5613 (64.7%)	
NAFLD, n (%)			<0.001
Yes	84 (1.8%)	394 (4.5%)	
No	4461 (98.2%)	8276 (95.5%)	
Lipid drug usage, n (%)			<0.001
Yes	0 (0.0%)	2232 (48.7%)	
No	1640 (100.0%)	2351 (51.3%)	
Coronary heart disease, n (%)			<0.001
Yes	318 (7.0%)	1995 (23.0%)	
No	4227 (93.0%)	6675 (77.0%)	
Heart failure, n (%)			<0.001
Yes	107 (2.4%)	672 (7.8%)	
No	4438 (97.6%)	7998 (92.2%)	
Sleep apnea, n (%)			<0.001
Yes	140 (3.1%)	662 (7.6%)	
No	4405 (96.9%)	8008 (92.4%)	

MHO, metabolically healthy obesity; MUHO, metabolically unhealthy obesity; T2D, type 2 diabetes; NAFLD, metabolic dysfunction-association fatty liver disease; HDL, high-density lipoprotein; HbA1c, glycosylated hemoglobin, type A1c. Continuous variables were compared using Student’s t-test, and categorical variables were compared using the chi-square test.

### 3.2 Identification of a six-metabolite signature associated with MHO

**Figure 2:**
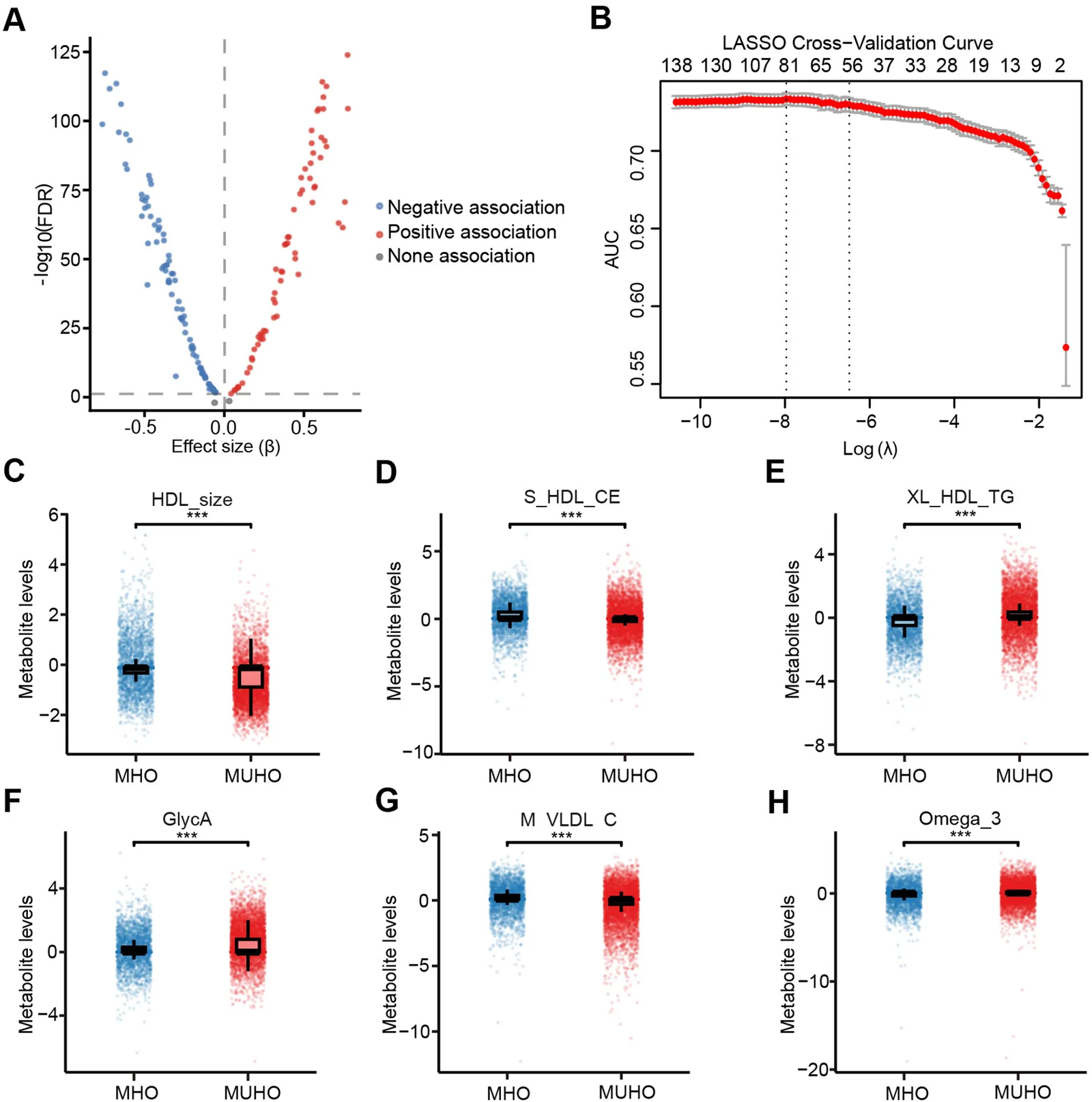
Identification of metabolomic signatures distinguishing MHO from MUHO. **(A)** Potential metabolites associated with MHO and MUHO identified by univariable logistic regression analysis. **(B)** Candidate metabolites selected based on LASSO regression analysis. **(C)** Comparison of HDL_size levels between the MHO and MUHO groups. **(D)** Comparison of S_HDL_CE levels between the MHO and MUHO groups. **(E)** Comparison of XL_HDL_TG levels between the MHO and MUHO groups. **(F)** Comparison of GlyCA levels between the MHO and MUHO groups. **(G)** Comparison of M_VLDL_C levels between the MHO and MUHO groups. **(H)** Comparison of Omega_3 levels between the MHO and MUHO groups. Asterisks represent statistical significance: *P< 0.05, **P< 0.001, and ***P< 0.0001.

To identify circulating metabolites associated with metabolic health status, we first performed metabolome-wide univariable logistic regression across 154 metabolites, using MHO status as the outcome. Among these metabolites, 152 were significantly associated with metabolic health status, including 87 negatively associated and 65 positively associated metabolites (**Figure 2A**; **Supplementary Table 1**). This broad pattern indicated that MHO and MUHO differed across multiple metabolic domains rather than by isolated biomarker alterations.

To reduce dimensionality and identify candidate metabolomic predictors, LASSO regression was then applied to the metabolite panel. This procedure identified 57 candidate metabolites with discriminatory value (**Figure 2B**; **Supplementary Table 2**). Following the screening principles outlined in the methods section, many metabolite indicators exhibited strong correlations and represented overlapping lipoprotein subclasses or metabolic processes. Therefore, candidate metabolites were further evaluated based on the magnitude of the LASSO coefficient, pairwise correlations, and biological domain coverage. Among highly correlated metabolites with similar biological interpretations, representative variables were retained to reduce redundancy and improve model simplicity. Ultimately, a combination of six metabolites was determined, including HDL_size, S_HDL_CE, XL_HDL_TG, GlycA, M_VLDL_C, and Omega_3 (**Supplementary Table 3**). These six metabolites captured distinct but complementary metabolic axes, including HDL particle structure, HDL lipid composition, triglyceride enrichment of HDL particles, VLDL-related lipid metabolism, and fatty acid composition. Group comparisons showed significant differences in six metabolites between MHO and MUHO (**Figures 2C–H**), supporting their relevance as representative markers of metabolic health status in obesity.

### 3.3 Metabolome-wide signatures distinguishing MHO and MUHO

**Figure 3:**
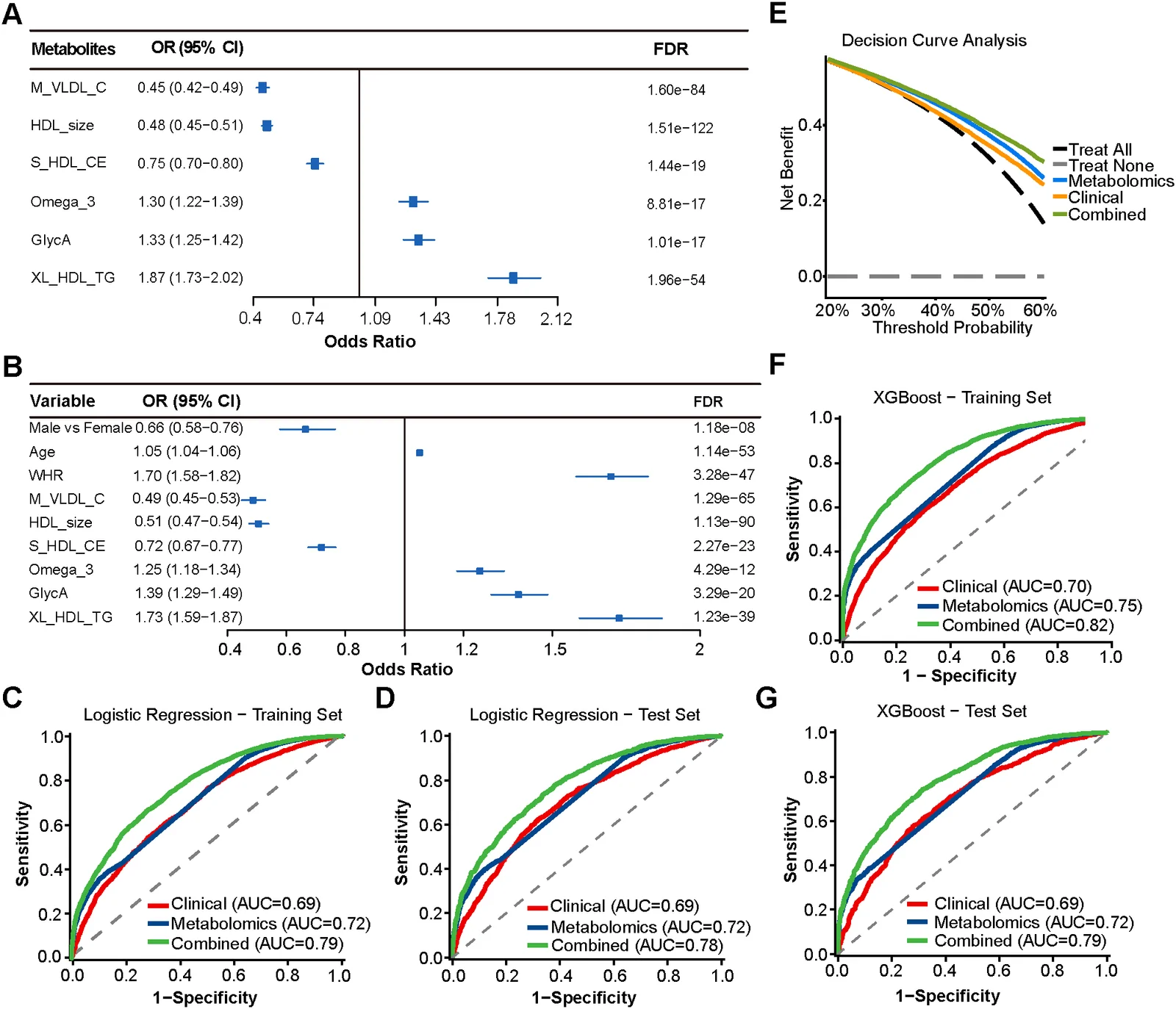
Discriminatory performance of clinical, metabolite, and combined clinical-metabolite models. **(A)** Forest plot showing the associations between individual metabolites in the metabolite model and MUHO, including OR, 95% CI, and P values. **(B)** Forest plot showing the associations of clinical variables and metabolites in the combined model with MUHO, including OR, 95% CI, and P values. **(C)** Comparison of ROC curves for the clinical model, metabolite model, and combined model constructed using logistic regression in the training set. **(D)** Comparison of ROC curves for the clinical model, metabolite model, and combined model constructed using logistic regression in the validation set. **(E)** Decision curve analysis of the clinical model, metabolite model, and combined model. **(F)** Comparison of ROC curves for the clinical model, metabolite model, and combined model constructed using XGBoost in the training set. **(G)** Comparison of ROC curves for the clinical model, metabolite model, and combined model constructed using XGBoost in the validation set.

We next evaluated whether the six-metabolite signature could distinguish MHO from MUHO. In the metabolites logistic regression model, all six metabolites were significantly associated with MUHO (**Figure 3A**). Among them, Omega_3, GlycA, and XL_HDL_TG were positively associated with MUHO and therefore represented risk-related metabolic features, whereas the remaining three metabolites were inversely associated with MUHO and represented protective metabolic features. After adjustment for age, sex, and waist-to-hip ratio, all metabolites remained independently associated with MUHO (**Figure 3B**; **Supplementary Table 4**), indicating that the metabolite signature captured obesity-related metabolic heterogeneity beyond conventional clinical variables.

Three classification models were developed, including a clinical model based on age, sex, and waist-to-hip ratio, a metabolite model based on the six-metabolite signature, and a combined model incorporating both clinical variables and metabolites. In the training set, the combined model showed the best discriminatory performance, with a higher AUC than the clinical and metabolite models, respectively (0.79 vs. 0.69 and 0.72; **Figure 3C**). Similar results were observed in the test set, with AUCs of 0.78, 0.69, and 0.72 for the combined, clinical, and metabolite models, respectively (**Figure 3D**), suggesting good internal robustness. Pairwise DeLong tests confirmed significant improvements in AUC for the metabolite model over the clinical model (ΔAUC = 0.023, P = 0.002) and for the combined model over both the clinical model (ΔAUC = 0.084, P< 0.001) and the metabolite model (ΔAUC = 0.061, P< 0.001) (**Supplementary Table 5**). Consistently, NRI and IDI analyses further supported the added predictive value of the six-metabolite signature for distinguishing MHO from MUHO. Calibration analysis demonstrated good agreement between predicted and observed outcomes. Bootstrap validation yielded an optimism-corrected AUC of 0.77. The Brier score was 0.18, the calibration intercept was close to 0, and the calibration slope was close to 1, indicating good calibration of the combined model.

Decision curve analysis further showed that the combined clinical-metabolomic model provided the greatest net benefit across a broad range of threshold probabilities (**Figure 3E**). To assess whether nonlinear modeling improved phenotype discrimination, XGBoost models were also constructed using the same feature sets. The XGBoost models showed comparable results, with the combined model consistently outperforming the clinical and metabolomic models alone in both training and test sets (**Figures 3F, G**). Together, these findings demonstrated that the six-metabolite signature provided stable and interpretable discriminatory value for distinguishing MHO from MUHO.

### 3.4 Sensitivity and subgroup analyses

To assess the robustness of the main findings, we performed several sensitivity and subgroup analyses. Under the strict definition of metabolic health, 1,609 participants were classified as MHO and 11,606 as MUHO. All six prespecified metabolites remained significantly associated with prevalent MUHO after adjustment for age, sex, and waist-to-hip ratio, with unchanged directions of association (**Supplementary Figure 1**). In the test set, the clinical, metabolomic, and combined models achieved AUCs of 0.69, 0.71, and 0.77, respectively, supporting the robustness of the six-metabolite signature across alternative definitions of metabolic health.

To examine whether phenotype discrimination was primarily driven by overlap between lipid-related metabolites and the lipid criteria used to define MUHO, we repeated the analysis after excluding HDL_size, S_HDL_CE, XL_HDL_TG, and M_VLDL_C. The reduced metabolomic model retained GlycA and Omega_3. In the test set, the GlycA–Omega_3 model achieved an AUC of 0.69, whereas the corresponding combined model achieved an AUC of 0.75 (**Supplementary Figure 2**), indicating that the distinction between MHO and MUHO was not entirely attributable to lipid-related metabolites.

We also constructed an expanded clinical model including age, sex, waist-to-hip ratio, BMI, smoking status, and alcohol consumption. In the test set, the expanded clinical model achieved an AUC of 0.68, compared with 0.69 for the primary clinical model. The expanded clinical–metabolomic model achieved an AUC of 0.78, comparable to that of the primary combined model, with no significant difference between them (**Supplementary Figure 3**). Thus, the inclusion of additional anthropometric and lifestyle variables did not materially alter the discriminatory contribution of the six-metabolite signature.

In sex-stratified analyses, the combined model showed broadly comparable performance in men and women, with test-set AUCs of 0.74 and 0.76, respectively (**Supplementary Table 6**). Calibration slopes and Brier scores were also similar between sexes. No significant interaction was observed between sex and the combined-model linear predictor (P for interaction = 0.21), indicating no material sex-related difference in the association between the model score and prevalent MUHO status.

Finally, to assess the potential influence of lipid-lowering therapy, we excluded 2,232 participants using lipid-lowering medication, leaving 10,983 participants. In this subset, the six-metabolite signature remained consistent with the primary findings. The clinical, metabolomic, and combined models achieved test-set AUCs of 0.65, 0.70, and 0.75, respectively (**Supplementary Figure 4**), suggesting that the observed phenotype discrimination was not materially driven by lipid-lowering medication use.

To evaluate whether highly ranked metabolites excluded from the final panel provided additional predictive information, we further examined LDL_P, S_HDL_P, and Phosphoglyc (**Supplementary Figure 5**). After adjustment for age, sex, and waist-to-hip ratio, LDL_P and S_HDL_P remained inversely associated with MUHO, whereas the association of Phosphoglyc was not statistically significant. However, incorporating these three metabolites into an expanded nine-metabolite model did not improve discriminatory performance. The expanded metabolomics model achieved AUCs of 0.72 and 0.71 in the training and test datasets, respectively, while the corresponding combined model achieved AUCs of 0.79 and 0.78, which were comparable to those of the original six-metabolite models. These findings supported retaining the more parsimonious six-metabolite panel.

### 3.5 Metabolomic risk score reveals hidden metabolic risk within MHO

To characterize metabolic heterogeneity within conventionally defined MHO, we calculated a weighted metabolomic signature score among the 4,545 participants with MHO. The score was derived from the six standardized metabolites weighted by their corresponding logistic regression coefficients: Metabolomic risk score = −0.740 × HDL_size + 0.626 × XL_HDL_TG − 0.290 × S_HDL_CE − 0.795 × M_VLDL_C + 0.265 × Omega_3 + 0.288 × GlycA + 0.862. The 25th and 75th percentiles of the score distribution were 0.365 and 0.675, respectively. Participants were therefore categorized into low-score (≤0.365; n=1,137), intermediate-score (>0.365 to ≤0.675; n=2,272), and high-score (>0.675; n=1,136) groups.

**Figure 4:**
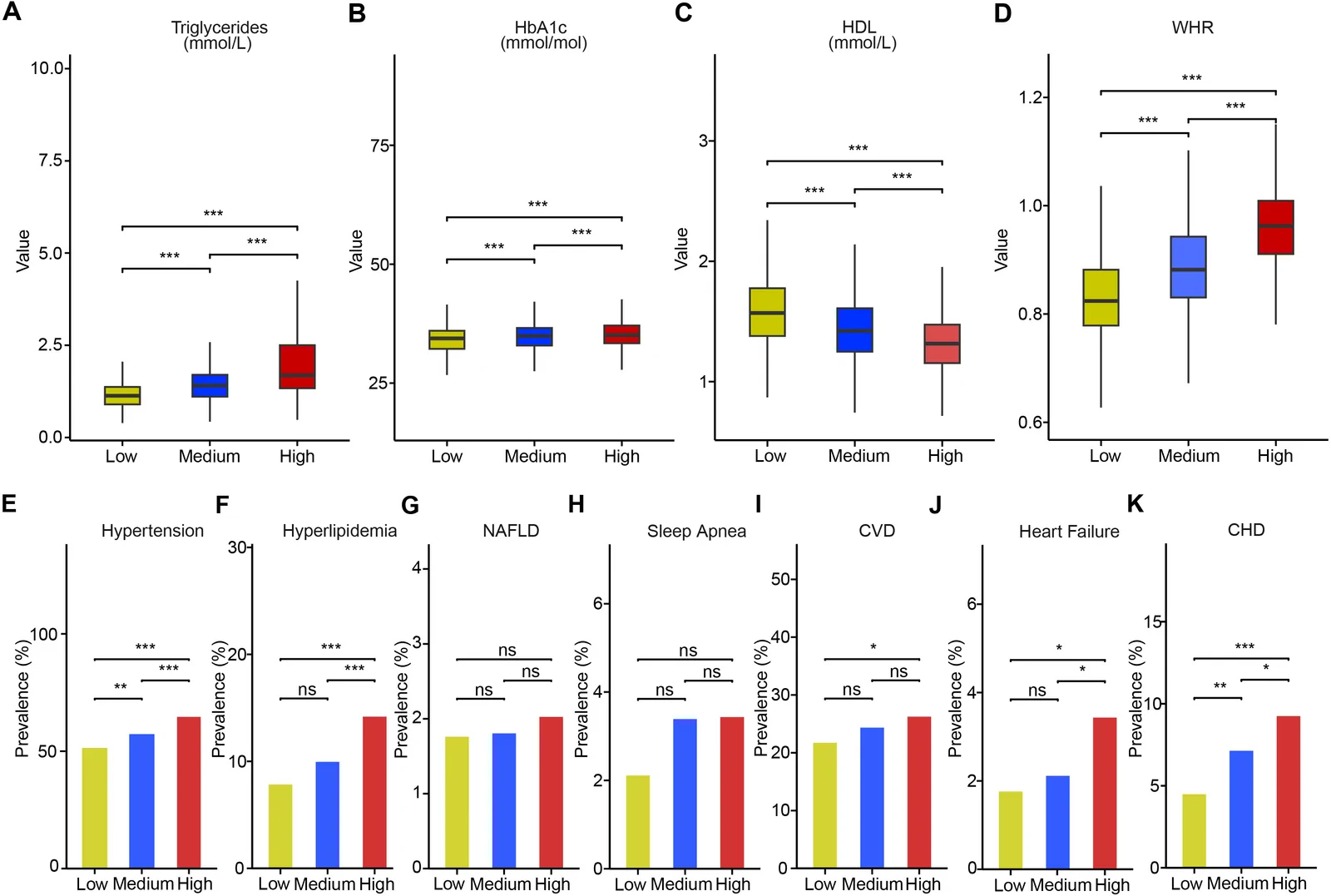
Metabolite signature identifies hidden high-risk phenotypes within MHO. Participants with metabolically healthy obesity were stratified into low-risk, intermediate-risk, and high-risk MHO subgroups according to the metabolite signature score. Clinical and cardiometabolic characteristics were compared across the three MHO subgroups, including **(A)** triglycerides, **(B)** HbA1c, **(C)** HDL, **(D)** WHR, **(E)** hypertension, **(F)** hyperlipidemia, **(G)** NAFLD, **(H)** sleep apnea, **(I)** CVD, **(J)** heart failure, and **(K)** CHD. Asterisks represent statistical significance: *P< 0.05, **P< 0.001, and ***P< 0.0001. HbA1c, glycated hemoglobin; HDL, high-density lipoprotein; NAFLD, nonalcoholic fatty liver disease; WHR, waist-to-hip ratio; CVD, cardiovascular disease; CHD, coronary heart disease.

Although all participants in this analysis met conventional criteria for MHO, marked clinical heterogeneity was observed across metabolite score groups. Higher metabolite risk scores were associated with progressively higher triglycerides (**Figure 4A**) and HbA1c (**Figure 4B**), together with lower HDL (**Figure 4C**) and higher waist-to-hip ratio (**Figure 4D**). These stepwise gradients indicate that the metabolite score captured residual variation in lipid metabolism, glycemic status, and central adiposity within individuals conventionally classified as MHO. Consistent with these metabolic differences, the prevalence of cardiometabolic comorbidities also varied across groups. Higher metabolite risk scores were associated with increased prevalence of hypertension (**Figure 4E**), hyperlipidemia (**Figure 4F**), NAFLD (**Figure 4G**), sleep apnea (**Figure 4H**), cardiovascular disease (**Figure 4I**), heart failure (**Figure 4J**), and coronary heart disease (**Figure 4K**). Among these, the gradients were particularly evident for hypertension, hyperlipidemia, and cardiovascular-related outcomes, whereas differences in NAFLD and sleep apnea appeared relatively modest.

Collectively, these findings suggest that conventionally defined MHO is not a homogeneous phenotype. The metabolite score identified hidden metabolic risk within MHO, characterized by adverse lipid metabolism, higher glycemic burden, and increased cardiometabolic comorbidity. Thus, the six-metabolite signature provided additional resolution for refining metabolic risk stratification beyond binary MHO/MUHO classification.

### 3.6 Proteomic analysis supports biological relevance of the metabolomic signature

**Figure 5:**
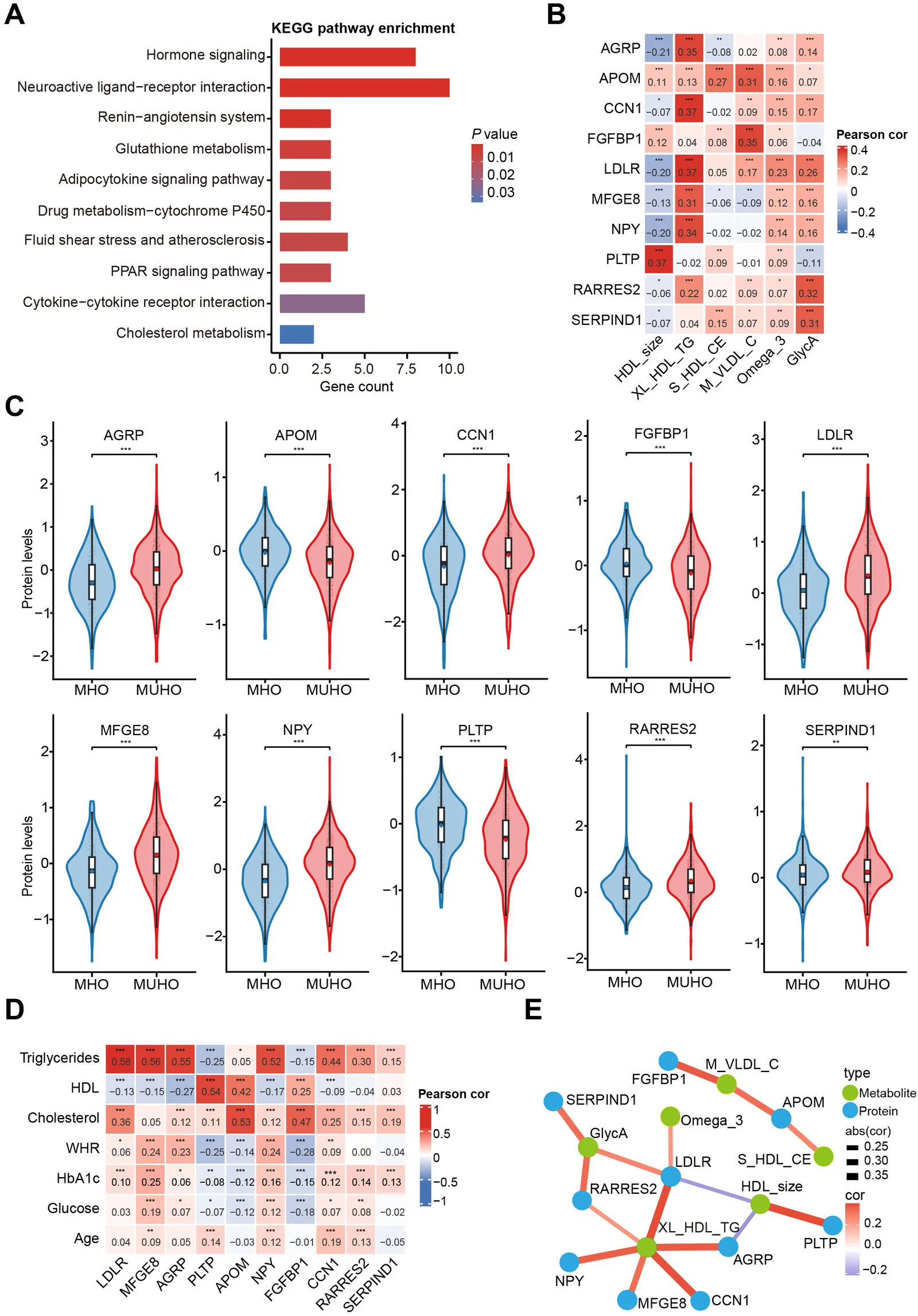
Integrative analysis of proteomic and metabolomic profiles. **(A)** KEGG pathway annotation of differentially expressed proteins between MHO and MUHO. **(B)** Correlation heatmap showing associations between differentially expressed proteins and the six metabolites. **(C)** Comparison of core protein levels between participants with MHO and MUHO. **(D)** Correlation heatmap showing associations between the six metabolites and clinical phenotypes. **(E)** Interaction network linking core proteins and the six metabolites. Asterisks represent statistical significance: *P< 0.05, **P< 0.001, and ***P< 0.0001.

To explore the biological context of the six-metabolite signature, we performed proteomic analysis in a subset of approximately 1408 participants with available protein data. Differential protein analysis identified 69 proteins with significantly different abundance between MHO and MUHO (**Supplementary Table 7**). KEGG enrichment analysis showed that these proteins were mainly involved in hormone signaling, neuroactive ligand-receptor interaction, renin-angiotensin system, glutathione metabolism, adipocytokine signaling, PPAR signaling, cytokine-related pathways, cholesterol metabolism, and vascular regulation (**Figure 5A**).

Pearson correlation analysis between the six metabolites and the 69 differential proteins identified ten metabolite-associated core proteins, including AGRP, APOM, CCN1, FGFBP1, LDLR, MFGE8, NPY, PLTP, RARRES2, and SERPIND1, based on an absolute correlation coefficient >0.30 and P< 0.01 (**Figure 5B**). These proteins showed significant abundance differences between MHO and MUHO, with distinct directionality across proteins (**Figure 5C**).

The ten core proteins were further associated with major metabolic traits, particularly triglycerides, HDL cholesterol, total cholesterol, waist-to-hip ratio, and HbA1c (**Figure 5D**). Network analysis revealed an interconnected metabolite-protein module linking the six-metabolite signature to lipid and cholesterol metabolism, adipokine signaling, inflammation and vascular regulation (**Figure 5E**; **Supplementary Table 8**). Together, these findings support the biological relevance of the six-metabolite signature and suggest that it reflects a broader molecular network underlying metabolic health heterogeneity in obesity.

## 4 Discussion

The principal finding of this study is that substantial metabolic heterogeneity exists even among individuals conventionally classified as metabolically healthy obesity (MHO). Using large-scale metabolomic profiling, we identified a six-metabolite signature that not only distinguished MHO from metabolically unhealthy obesity (MUHO), but also revealed hidden metabolic risk within MHO. Individuals with higher metabolomic risk scores exhibited a more adverse metabolic profile despite meeting conventional MHO criteria. These findings suggest that metabolic health in obesity may be better represented as a continuum of biological risk rather than a binary phenotype and support the use of molecular profiling to refine obesity risk stratification.

Most previous studies have relied on clinical definitions of metabolic health based on metabolic syndrome components. In contrast, our study demonstrates that metabolomic profiling can identify substantial biological heterogeneity beyond conventional clinical classifications. This provides a molecular framework for refining obesity phenotyping. The six-metabolite signature has clear biological interpretability. HDL_size and S_HDL_CE reflect HDL particle structure and cholesteryl ester content, both of which are relevant to HDL maturation, lipid transport, and reverse cholesterol transport (21, 22). XL_HDL_TG may indicate triglyceride enrichment of HDL particles, a feature associated with altered lipid exchange and impaired HDL function (23). M_VLDL_C reflects VLDL-related lipid transport and atherogenic lipoprotein metabolism (24). These lipid-related features are consistent with the concept that HDL quantity alone is insufficient to capture HDL function and that HDL remodeling may be closely related to cardiometabolic dysfunction (25, 26). GlycA, a composite NMR-based metabolic biomarker derived from glycosylated acute-phase proteins, reflects systemic inflammation and has been associated with chronic inflammatory and cardiometabolic risk states (27, 28). Omega_3 captures fatty acid composition and may reflect inflammatory, lipid, and vascular regulation (29, 30). Together, these six metabolites suggest that MUHO is not driven by one isolated abnormality, but by coordinated changes in lipoprotein remodeling, systemic inflammation, fatty acid biology, and vascular risk.

The classification analyses quantified the ability of the six-metabolite signature to distinguish MHO from MUHO. The combined model achieved the highest discrimination, and the DeLong, NRI, and IDI analyses indicated that metabolomic information provided additional discriminatory value beyond basic clinical variables. XGBoost did not materially outperform logistic regression, suggesting that a simpler and more interpretable model may be sufficient for integrating the selected clinical and metabolomic features. These analyses were intended to evaluate cross-sectional phenotype discrimination rather than to establish a prospective clinical prediction tool.

The most important conceptual contribution of this study is the identification of substantial metabolic heterogeneity within MHO. Previous studies have emphasized that MHO is variably defined, biologically complex, and not necessarily a stable or benign condition (31, 32). Meta-analyses and large cohort studies have shown that individuals with MHO may still have a higher long-term risk of cardiovascular disease and mortality than metabolically healthy normal-weight individuals (33), while longitudinal studies suggest that many transition to metabolically unhealthy states over time, with adverse cardiovascular consequences (34, 35). Our findings complement these observations by demonstrating cross-sectional metabolomic and clinical heterogeneity within MHO. Higher metabolomic signature scores were associated with less favorable lipid, glycemic, and adiposity profiles, as well as a greater prevalence of cardiometabolic comorbidities, indicating that some individuals classified as MHO exhibit characteristics overlapping with MUHO. From a translational perspective, metabolomics may complement conventional clinical assessment by capturing coordinated, systems-level metabolic remodeling that is not fully reflected by individual clinical markers (36). A concise and interpretable metabolite panel may therefore be more practical for population-level phenotyping than high-dimensional profiling, while computational approaches such as logistic regression and XGBoost can support the integration of clinical and molecular data; broader AI applications in disease detection and clinical decision support illustrate this potential (37, 38). However, because the present analysis was cross-sectional, we cannot determine whether these individuals are more likely to deteriorate metabolically or transition to MUHO over time.

An important consideration is that several components of the six-metabolite signature, particularly HDL_size, S_HDL_CE, XL_HDL_TG, and M_VLDL_C, are biologically related to the triglyceride and HDL-C criteria used to define MUHO. Thus, part of the discriminatory performance of the primary model may reflect outcome–predictor overlap. However, after excluding these lipid-related metabolites, a reduced model based on GlycA and Omega_3 retained meaningful discrimination, with a test-set AUC above 0.75 when combined with clinical variables. This finding suggests that the model captured information beyond the lipid criteria embedded in the outcome, potentially involving systemic inflammation and fatty acid composition. Overall, the observed metabolomic gradients support the view that metabolic health in obesity is heterogeneous and may be better represented as a continuum rather than as a strictly binary phenotype.

The definition of MHO remains debated, and different criteria identify overlapping but non-identical population subgroups. Our primary definition allowed no more than one metabolic abnormality because it reflected the commonly used absence-of-metabolic-syndrome framework and was suitable for evaluating residual heterogeneity within conventionally classified MHO. Nevertheless, this definition should not be interpreted as indicating the complete absence of metabolic impairment. In sensitivity analyses requiring zero metabolic abnormalities, the directions of association for the six metabolites and the incremental performance of the combined model were broadly consistent, supporting the robustness of the principal findings. However, the stricter definition identified a smaller and more selected MHO subgroup, highlighting that estimates of MHO prevalence and model performance remain definition-dependent.

This finding also reinforces the limitation of BMI and threshold-based metabolic definitions. In our cohort, BMI differed only modestly between MHO and MUHO, whereas waist-to-hip ratio and metabolic biomarkers showed more pronounced differences. This agrees with epidemiological evidence showing that central and abdominal adiposity are more strongly associated with cardiometabolic risk than total adiposity alone (39, 40). Mechanistically, visceral and ectopic fat depots are closely linked to insulin resistance, dyslipidemia, inflammation, and cardiometabolic disease (41, 42). A continuous metabolomic signature score may therefore provide a more biologically informed framework for characterizing heterogeneity within conventional obesity phenotypes.

The proteomic findings further strengthened the biological credibility of the six-metabolite signature. Differential proteins between MHO and MUHO were enriched in pathways related to adipocytokine signaling, PPAR signaling, cholesterol metabolism, renin-angiotensin signaling, and vascular regulation. These pathways are directly relevant to obesity-related inflammation, lipid handling, insulin resistance, and vascular dysfunction (43–45). Several core proteins identified in the metabolite-protein network are mechanistically plausible. APOM is mainly carried by HDL and contributes to lipid transport and sphingosine-1-phosphate biology (46). LDLR plays a central role in LDL clearance and cholesterol homeostasis (47). PLTP regulates phospholipid transfer, HDL remodeling, and atherosclerosis-related lipoprotein metabolism (48). RARRES2 encodes chemerin, an adipokine associated with obesity, inflammation, insulin resistance, and metabolic syndrome (49). Thus, the six-metabolite signature is unlikely to be merely a classifier, rather, it appears to reflect a coordinated proteomic-metabolomic network involving lipid transport, inflammation, adipocytokine signaling, and vascular dysfunction.

This study has several strengths. It was based on a large, deeply phenotypic UK Biobank cohort with standardized NMR-based metabolomic profiling. The analytical workflow integrated feature selection, model validation, risk stratification, and proteomic interpretation, yielding a concise and biologically interpretable six-metabolite signature with stable performance in the test set. Proteomic integration further provided molecular support by linking the signature to disease-relevant pathways. Several limitations should also be acknowledged. Although UK Biobank is a prospective cohort, the present analyses were primarily cross-sectional; therefore, we could not determine whether the metabolite score predicts future transition from MHO to MUHO or incident cardiometabolic events. In addition, a further limitation of this study is the lack of external validation. Although the six-metabolite signature showed stable performance through independent test-set and bootstrap validation, all analyses were conducted within the UK Biobank. Therefore, validation in independent cohorts with different ethnic backgrounds and metabolomic platforms is warranted before clinical application.

In conclusion, this study demonstrates that MHO and MUHO are characterized by broad metabolomic differences and that a concise six-metabolite signature can distinguish these phenotypes with good performance. More importantly, this signature reveals hidden cardiometabolic risk within conventionally defined MHO, supporting a molecular risk-spectrum view of obesity. Proteomic integration further suggests that the signature reflects coordinated changes in lipoprotein remodeling, inflammation, adipocytokine signaling, and vascular biology. These findings challenge the assumption that conventional MHO is uniformly healthy and support metabolomics-based refinement of obesity risk stratification.

## 5 Conclusions

In conclusion, we identified a six-metabolite signature that effectively distinguished MHO from MUHO and provided additional predictive value beyond conventional clinical variables. More importantly, when applied within individuals conventionally classified as MHO, the metabolomic signature revealed substantial heterogeneity and identified a subgroup characterized by adverse lipid profiles, higher glycemic burden, and increased metabolic comorbidity prevalence. These findings suggest that metabolic health in obesity is not adequately captured by current binary clinical classifications and may be better represented as a continuum of metabolic risk. Integrative proteomic analyses further supported the biological relevance of the metabolomic signature and linked it to pathways involved in lipoprotein remodeling, inflammation, and vascular regulation. Together, our findings demonstrate that metabolomics can uncover hidden metabolic risk within conventionally defined MHO and provide a molecular framework for refining obesity phenotyping and risk stratification. Future longitudinal studies are warranted to determine whether metabolomics-defined high-risk MHO individuals are at increased risk of adverse clinical outcomes.

## Data Availability

The original contributions presented in the study are included in the article/**Supplementary Material**. Further inquiries can be directed to the corresponding authors.

## Supplementary material

Supplementary Figure 1 Sensitivity analysis using a strict definition of metabolic health. **(A)** Forest plot showing the associations of clinical variables and metabolites in the combined model with MUHO. **(B)** Forest plot showing the associations between individual metabolites in the metabolite model and MUHO, including ORs, 95% CIs, and P values. **(C)** Comparison of ROC curves for the clinical, metabolite, and combined models constructed using logistic regression in the training set. **(D)** Comparison of ROC curves for the clinical, metabolite, and combined models constructed using logistic regression in the test set.

Supplementary Figure 2 Sensitivity analysis excluding lipid-related metabolites. **(A)** Comparison of ROC curves for the clinical, reduced metabolomic, and corresponding combined clinical-metabolomic models constructed using logistic regression in the training set. **(B)** Comparison of ROC curves for the clinical, reduced metabolomic, and corresponding combined clinical-metabolomic models constructed using logistic regression in the test set.

Supplementary Figure 3 Discriminatory performance of the expanded clinical model. **(A)** Forest plot showing the associations of variables included in the expanded clinical model-age, sex, waist-to-hip ratio, BMI, smoking status, and alcohol consumption-with MUHO. **(B)** Comparison of ROC curves for the expanded clinical, metabolomic, and expanded combined clinical-metabolomic models constructed using logistic regression in the training set. **(C)** Comparison of ROC curves for the expanded clinical, metabolomic, and expanded combined clinical–metabolomic models constructed using logistic regression in the test set.

Supplementary Figure 4 Sensitivity analysis excluding participants using lipid-lowering medication. The clinical, metabolomic, and combined clinical–metabolomic models were refitted using the same prespecified six-metabolite panel. **(A)** Comparison of ROC curves for the three models in the training set. **(B)** Comparison of ROC curves for the three models in the test set. AUC, area under the curve; ROC, receiver operating characteristic; MHO, metabolically healthy obesity; MUHO, metabolically unhealthy obesity.

Supplementary Figure 5 Associations and discriminatory performance of major candidate metabolites excluded from the final six-metabolite panel. **(A)** Multivariable logistic regression analysis showing the associations of sex, age, waist-to-hip ratio, S_HDL_P, LDL_P, and Phosphoglyc with metabolically unhealthy obesity (MUHO) in the training dataset. **(B)** Associations of the three major excluded candidate metabolites-LDL_P, S_HDL_P, and Phosphoglyc—with MUHO after adjustment for sex, age, and waist-to-hip ratio. Receiver operating characteristic curves for the clinical, metabolomic, and combined models in the training dataset **(C)** and independent test dataset **(D)**. The clinical model included sex, age, and waist-to-hip ratio; the metabolomic model included the expanded nine-metabolite panel consisting of the six selected metabolites plus LDL_P, S_HDL_P, and Phosphoglyc; and the combined model included both clinical variables and the expanded metabolomic panel.

Supplementary Table 1 Results of univariable logistic regression analysis of metab-olites associated with MHO and MUHO.

Supplementary Table 2 Candidate metabolite predictors identified by LASSO regression analysis.

Supplementary Table 3 Selection process, functional classification, and correlation structure of candidate metabolites.

Supplementary Table 4 Logistic regression model incorporating clinical variables and the six-metabolite signature.

Supplementary Table 5 Pairwise DeLong test results for model performance comparisons.

Supplementary Table 6 Sex-stratified discriminatory performance of the combined model.

Supplementary Table 7 Differentially expressed proteins between MHO and MUHO.

Supplementary Table 8 KEGG pathway enrichment analysis of differentially expressed proteins.
